# New Frontiers for the NFIL3 bZIP Transcription Factor in Cancer, Metabolism and Beyond

**DOI:** 10.15190/d.2014.7

**Published:** 2014-06-30

**Authors:** Megan Keniry, Robert K. Dearth, Michael Persans, Ramon Parsons

**Affiliations:** Department of Biology, University of Texas- Pan American, 1201 W. University Dr., Edinburg, TX 78539, USA; Department of Oncological Sciences, Icahn School of Medicine at Mount Sinai, 1470 Madison Ave HCSM 6-117, New York, NY 10029, USA

**Keywords:** NFIL3, CEBP, CEBPbeta, Metabolism, Cancer, Immunology

## Abstract

The bZIP transcription factor NFIL3 (Nuclear factor Interleukin 3 regulated, also known as E4 binding protein 4, E4BP4) regulates diverse biological processes from circadian rhythm to cellular viability. Recently, a host of novel roles have been identified for NFIL3 in immunological signal transduction, cancer, aging and metabolism. Elucidating the signaling pathways that are impacted by NFIL3 and the regulatory mechanisms that it targets, inhibits or activates will be critical for developing a clearer picture of its physiological roles in disease and normal processes. This review will discuss the recent advances and emerging issues regarding NFIL3-mediated transcriptional regulation of CEBPb and FOXO1 activated genes and signal transduction.

## **SUMMARY**

1. Introduction
2. NFIL3 is a b-ZIP Transcriptional Regulator
3. Nfil3 Regulates Circadian Rhythm
4. Light and Nutrient Availability Regulate *NFIL3* Expression
5. NFIL3 in Development and Cell fate
  - 5.1 Immunological Role of Nfil3
  - 5.2 NFIL3 is Involved in Heart Development and Aging
6. NFIL3 Influences Cellular Survival
7. NFIL3 in Cancer
8. Nfil3 in Neuronal Regeneration
9. Nfil3 in Osteoblast Signal Transduction
10. NFIL3-CEBPb Antagonism: A Recurring Theme
11. Conclusions and Future Directions

## **1.** **Introduction**

The NFIL3 transcriptional repressor impacts many cellular processes and is widely expressed in normal murine and human tissues^1-19^. Often, NFIL3 action opposes that of transcriptional activators by competing for access to target sites on DNA^2,7,13,15,20-22^. NFIL3 is also commonly found within the context of feedback regulatory circuits^21^. The list of physiological roles for NFIL3 has evolved tremendously over the last few years. In addition to roles in circadian rhythm and B cell/neuronal survival, NFIL3 is now implicated in having far reaching activities from immunological signal transduction to metabolism, aging and cancer^1-4,6,7,9-11,14,18,23,24^. Here, we give an overview of NFIL3 transcriptional regulatory circuits and discuss emerging areas of NFIL3 research regarding cancer and diabetes.

## **2.** **NFIL3 is a b-ZIP Transcriptional Regulator**

**Figure 1:**
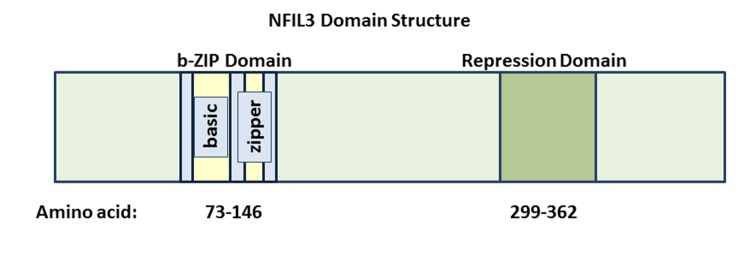
NFIL3 is a bZIP Transcriptional Repressor This schematic depicts the overall domain structure of the NFIL3 transcriptional repressor. The bZIP and minimal repressor domains are indicated.

NFIL3 was initially identified by its ability to bind to and repress an E4 promoter sequence containing an ATF consensus site^25,26^. Several years later, NFIL3 was shown to bind to and activate the transcription of an *IL3* (*Interleukin 3*) promoter sequence^27^. Although most of the published biological activities for NFIL3 involve its ability to repress transcription, a handful of mammalian cell culture studies as well as murine models indicate that it may also activate transcription by novel mechanisms^27,28^. The 462 amino acid sequence of NFIL3 includes a b-ZIP domain spanning amino acids 73-146 (**Figure 1**). The N-terminal portion of this domain (amino acids 79-95) contains the basic motif, which directly binds to DNA. The C-terminal portion of the b-ZIP domain (amino acids 99-106) contains an amphipathic leucine zipper region, which is responsible for homo-dimerization as well as hetero-dimerization interactions^29^. The NFIL3 protein also has a unique transcriptional repression domain that spans amino acids 299-363. This repressor domain function is transferable, since its fusion with the GAL4 DNA binding domain leads to transcriptional repression in reporter assays^30^. The molecular mechanisms employed by NFIL3 to repress transcription remain to be fully elucidated. In cancer cells, NFIL3 physically associates with Histone deacetylase 2 (HDAC2) to repress the *TRAIL (Tumor Necrosis Factor Ligand Superfamily, Member 10), FAS (TNF receptor superfamily member 6)* and *GADD45a (Growth Arrest and DNA-damage-inducible, alpha)* genes^2^. In hepatocytes Nfil3 represses *Fgf21 (Fibroblast Growth Factor 21)* expression in a histone methyltransferase G9a-dependent manner^31^. Nfil3 represses many additional genes including *Period 2 (Per2), Arntl (Aryl Hydrocarbon Receptor Nuclear Translocator-Like), Usp2-45 (Ubiquitin-specific protease 2-45), BGL-GS* (also known as BCL2*-like 14), Cox2 (Cyclooxygenase 2), Runx2 (Runt-related transcription factor 2), Phex (Phosphate Regulating Endopeptidase Homolog), Ptgs2 (Prostaglandin-endoperoxide synthase 2*, also known as *Cox2), Pgr (Progesterone receptor),* and *Areg (amphiregulin)*, which are regulated in diverse tissues ranging from pancreatic beta cells to osteoblasts^21,32-36^.

## **3.** **Nfil3 Regulates Circadian Rhythm**

The *Nfil3* gene is expressed in a circadian manner and encodes a regulator of circadian rhythm^20,24,37-39^. Organisms ranging from bacteria to humans have endogenous clocks or circadian rhythms that integrate intrinsic and extrinsic cues to optimize cellular growth and survival. These clocks are regulated by complex, interlocked feedback loops that are, in part, transcriptionally directed^37^. The three most common circadian transcription factor consensus sites are: **1)** E/E’ box elements which are activated by the Clock (circadian locomotor output cycles kaput)/Bmal1 (brain and muscle ARNT-like protein 1) protein dimer **2)** Rev-ErbA/ROR elements (RREs) which are repressed by NR1D1 (Nuclear Receptor subfamily 1, group D, member 1, also known as Rev/ErbA) and **3)** D box elements which are activated by the bZIP transcriptional activator Dbp (D site albumin promoter binding protein) and repressed by the bZIP transcriptional repressor Nfil3^37^. The mammalian circadian rhythm is initiated by the basic helix loop helix (bHLH)-PAS transcription factors Clock and Bmal1 which form heterodimers that induce the transcription of genes containing E box promoter elements including Period genes (*Per1*, *Per2 *and *Per3*), Cryptochrome genes (*Cry1* and *Cry2*), *Dbp*, and *Rev/ErbA*^40,41^. Transcriptional programs that activate genes in circadian rhythm are eventually turned off by negative feedback mechanisms. Period and Cry proteins form heterodimers to ultimately hinder the induction of E box regulated genes, Rev/ErbA repress RRE regulated genes including the *Bmal1* gene and NFIL3 represses D box regulated genes^40,41^. The* Nfil3 *promoter**contains numerous RREs thereby facilitating its circadian expression^37^. Mammalian *Nfil3* shows circadian expression in a large number of tissues including suprachiasmatic nuclei, liver, kidney, aorta, skeletal muscle, adrenal gland, and adipose tissue^42^.

**Figure 2:**
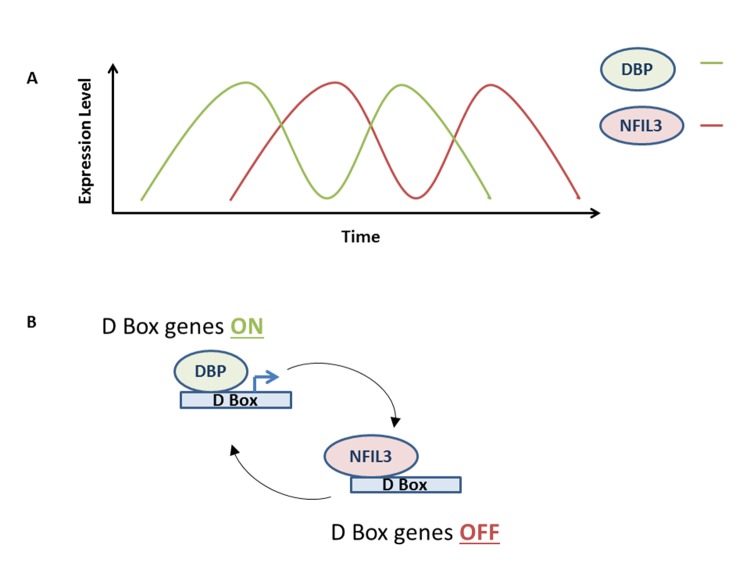
NFIL3 regulates D-box genes anti-phase to DBP **(A)** Schematic shows how NFIL3 and DBP peak at different times during the circadian rhythm. **(B)** DBP binds to and activates D-box genes, whereas NFIL3 binds to and represses D-box genes.

The mode of NFIL3 action on circadian rhythm may be prototypical of its action on other cellular processes. In circadian regulation, the NFIL3 repressor acts in an anti-phase manner with respect to the bZIP transcriptional activator DBP (**Figure 2**)^20,37^. NFIL3 and DBP compete for access to D box elements, exerting opposite effects on target genes^20^. NFIL3 expression peaks when DBP expression is at its lowest and *vice versa* (**Figure 2** [A]). Therefore, D box regulated genes are repressed when levels of NFIL3 are high and are induced (by DBP) when NFIL3 levels are low (**Figure 2** [B]). One of the most thought provoking roles of Nfil3 action in the circadian rhythm is that it has recently been shown to regulate period length (the time to complete a circadian cycle, which is normally 24 hours) in Rat1 fibroblast cells^38^. Specifically, the loss of Nfil3 lengthens period length whereas the overexpression of Nfil3 shortens period length. Levels of Dbp have opposite effects on period length^38^. Frequently, the circadian rhythm is coupled to cellular processes such as cell division and metabolism^43-50^. It would be informative to determine whether Rat1 cells grow more quickly with exogenous *Nfil3* expression as one would predict with the altered circadian period length. In addition, with a shorter period, are all stages of the circadian rhythm and related cellular processes affected equally by exogenous *Nfil3* or are certain aspects of this network differentially impacted? Finally, it will be important to determine whether NFIL3 affects period length in humans and mice.

Another recent discovery links the circadian function of Nfil3 to the development of Interleukin 17 producing CD4+ helper T (T_H_17) cells in the immune system^51^. Th17 cells protect organisms from bacterial and fungal infections on mucosal membranes and are also associated with inflammatory disease^52,53^. Nfil3 was found to suppress T_H_17 cell development by binding to the promoter of the orphan nuclear receptor *Rorgt *gene**and repressing transcription; Rorgt is required for the specification of T_H_17 cells^51,54^. Interestingly, *Nfil3* was found to be highly expressed at night in mice whereas *Rorgt* was found highly expressed during the day^51^. Accordingly, T_H_17 cell frequencies were significantly higher during the day in with-type mice^51^. This diurnal difference in T_H_17 cells was abrogated in *Nfil3* -/- mice^51^. This study is an exciting example that links the circadian and immunological functions of Nfil3. Overall, these emerging areas of Nfil3 regulated circadian processes highlight not only the basic mechanistic insights of its action, but also important connections within a broader cellular context.

## **4.** **Light and Nutrient Availability Regulate NFIL3 Expression**

The circadian rhythm has been shown to be coupled to metabolic processes in a number of tissues to coordinate behaviors such as rest and locomotion with cues such as light and nutrient availability^55-57^. As a circadian regulator, Nfil3 impacts metabolism by being part of the cell intrinsic oscillator^20-37^. In addition to cell intrinsic regulation, Nfil3 stands out as a circadian component that is induced by environmental cues such as light and feeding, thereby enabling cells to integrate internal and external cues^24,58^. The chick pineal gland is used as a model system to study circadian oscillations in response to light due to the fact that chick pinealocytes possess a circadian oscillator and a photo-transduction pathway for light entrainment^57^. By exposing young chicks to varying light/dark cycles, it was found that NFIL3 had a role in light entrainment. Under normal conditions, *NFIL3* expression in the chicken pineal gland peaks at early subjective night^32^. NFIL3 protein represses the expression of the *PERIOD2* (*PER2*) gene, which is expressed in an anti-phase manner to NFIL3 and peaks in the morning^32^. Light exposure during early subjective night leads to an induction in *NFIL3* gene expression and a delay in the expression of *PER2* the next morning. Sterol Regulatory Element-Binding Protein (SREBP) is a transcription factor that induces *NFIL3* expression in the chick pineal gland upon light exposure during early subjective night to ultimately cause a delay in *PER2* expression the next morning^58^. In sum, studies in chicks have revealed a role for Nfil3 in shifting the expression of *PER2* to a later time in response to light. It will be interesting to determine whether related mechanisms are employed in mammals.

Insulin and feeding are additional environmental cues that induce the expression of *Nfil3*. Feeding potently induces the expression of *Nfil3* in the mouse liver, whereas fasting has the opposite effect^24^. Furthermore, insulin induces *Nfil3* expression in Hepa1C1C-7 cells in a PI3K dependent manner. One of the Nfil3 repressed genes in the liver is *Fgf21* encodes a potent anti-diabetic and triglyceride lowering hormone^24^. During fasting, Fgf21 is critical for lipolysis, gluconeogenesis, and ketogenesis. Mechanistically, Nfil3 physically associates with D box elements on the *Fgf21* promoter in hepatocytes to repress transcription in a manner that depends on the histone methyltransferase G9a^31^. Thus, upon nutrient availability, Nfil3 action may lead to an epigenetic shift to maximize metabolism and biogenesis. Additionally, *Ubiquitin-specific protease 2-45* (*Usp2-45*) was identified as another Nfil3 repressed gene in hepatocytes^34^. *Usp2-45* encodes a deubiquitinase that regulates gluconeogenesis and glucose metabolism in the liver^34^. Under starvation conditions, Peroxisome proliferator-activated receptor-gamma coactivator alpha (Pgc1a) and Peroxisome proliferator-activated receptor-gamma coactivator beta (Pgc1b) along with Hepatocyte nuclear factor 4 (Hnf4) activate the transcription of *Usp2-45*, whereas Nfil3 strongly represses this gene under fed conditions. The ability of Pgc1a to induce *Usp2-45* is strongly enhanced when cells are transduced with *Nfil3* shRNA (to diminish *Nfil3* expression)^34^. In summary, under fed conditions, *Nfil3* expression is induced, leading to the repression of a number of genes (*Fgf21* and *Usp2-45*) to shift metabolic processes. Taken as a whole, the ability of Nfil3 to regulate metabolism is becoming increasingly evident and suggests that it may have a role in diabetes.

## **5.** **NFIL3 in Development and Cell Fate**

A number of breakthrough studies have revealed that NFIL3 not only regulates oscillatory mechanisms, but also has tremendous impacts on developmental processes. Some of the most significant advances relating to NFIL3 biology were recently made in the field of immunology. Nfil3 was recently found to be essentially required for Natural Killer (NK) cell development and function. Nfil3 has also been found to impact B-cell, T-cell, dendritic cell and macrophage responses. In addition to roles in immunological development, NFIL3 has also been recently found to significantly impact heart development and aging in numerous model organisms. These studies highlight completely novel roles for NFIL3 in the field of developmental biology.

### ***5.1 Immunological Role of Nfil3*****

Recently it has become clear that Nfil3 has significant contributions to immunological development and function^59^. Initially shown to be critical for natural killer cell (NK) development, Nfil3 is also required for efficient Immunoglobulin E (IgE) class switching and for the attenuation of numerous inflammatory responses^1,6,12,14,23^. Arguably, the most striking immunological phenotype for mice that lack *Nfil3* is the dramatic loss of mature NK cells^14,23^. Mice that were *nfil3* -/- had normal B and T cell development, but showed defective development, maturation and function of NK cells. A 35 fold reduction of splenic NK cells was observed in *nfil3* -/- mice. The few NK cells that did develop in *nfil3* -/- mice were functionally defective in the ability to stimulate IFN-g (Interferon Gamma) upon IL2 (Interleukin 2) plus IL12 (Interleukin 12) stimulation. Of note, Nfil3 was recently found to be dispensable for the development of TRAIL+ NK cells^60^. Therefore, Nfil3 is not required for the development of all NK lineages. NK cells are best known for their cytotoxicity to stressed cells such as those that are virally infected or cancerous. NK cells also have important roles in immunological modulation. For example, NK cell action can dampen CD8+ T cell immune response to viruses, leading to chronic infections as seen with HIV and Hepatitis B^61^. A unique set of human decidual natural killer cells have recently been shown to express NFIL3 and have important roles in tissue remodeling, neoangiogenesis, and immune modulation to prevent fetal rejection^62^. These studies highlight the central role of Nfil3 in NK development and suggest that it has an impact in defending organisms against viral infections.

Aside from NK phenotype, Nfil3 also has roles in B cell, dendritic cell, T cell and macrophage-derived immunological responses^59^. In murine B cells, Nfil3 is required for IgE class switching and thus generation of IgE production, involved in allergic response^11,12^. Nfil3 binds to the Ie exon promoter, which is thought to promote the production of IgE^12^. Mice that are *nfil3* -/- also lack mature CD8a+ dendritic cells that are crucial for antigen presentation and cross priming CD8+ T cells against cell presented antigens^10^. Other Nfil3 immunological events appear to involve potential feedback loops where Nfil3 may have roles in attenuating inflammation by either inhibiting the production of cytokines such as IL12 in macrophages or IL5 (Interleukin 5) and IL13 (Interleukin 13) in helper Th2 cells^6,9,10,63^. Nfil3 also augments the expression of the anti-inflammatory cytokine IL10 (Interleukin 10) in helper T cells (Th1 and Th2)^28^. Interestingly, *NFIL3* is highly expressed in T cells that were isolated from patients with systemic lupus erythematosus (SLE); studies using human T cells showed that exogenous *NFIL3* hindered T-cell activation and self-reactivity^64^. Overall, *nfil3*-/- mice are immunologically deficient with an almost complete absence of NK function, highly defective IgE production and defective antigen presentation from CD8+ T cells. These mice also lack important immunological regulatory mechanisms to hinder inflammation such as the production of IL10.

### ***5.2 NFIL3 is Involved in Heart Development and Aging***

The ability of NFIL3 to influence heart development and function has recently emerged. In *Drosophila melanogaster*, transcriptome analysis revealed that aged hearts express more of the NFIL3 homolog *Vrille* than younger hearts^5^. Aging in *Drosophila* heart tissue is characterized by an increase in heart period (HP; average time between successive end-diastolic positions). *Vrille* over-expression led to dilation of the heart. The loss of *Vrille* dramatically influenced HP: normal flies show a 55% increase in HP between ages 10 and 45 days, whereas *Vrille* loss of function flies only showed a 6% increase^5^. A significant number of the putative Vrille target genes, based on the presence of consensus sites and loss of expression in aged flies, were involved in mitochondrial function. Therefore, Vrille may hinder mitochondrial function to promote heart aging.

NFIL3 has also recently been investigated as having a role in heart development and disease in zebra fish (*Danio rerio*) and rats (*Rattus norvegicus)*. Specifically, loss-of-function analyses of the NFIL3 homolog in zebra fish indicate that it is important for normal heart development^65^. RNAi targeting of the zebra fish NFIL3 homolog led to malformed looping of the embryonic heart tube which occluded blood flow and retarded cardiac growth^65^. This same study showed that the over-expression of *Nfil3* in rat embryonic fibroblasts induced the expression of survival genes such as *Igf1* (*Insulin-like growth factor 1*), *Igf1r* (*Insulin-like growth factor 1 receptor*) and *Bcl2* (*B-cell lymphoma 2*) and hindered caspase 3 induction preventing apoptosis^65^. Yet another study showed that infarct volume and fibrosis were higher in mouse models subjected to ischemia/reperfusion during the time of day when *Nfil3* was at its lowest, suggesting a survival role for *Nfil3* in the heart^66^. Altogether, Nfil3 is emerging as an important cardiac signaling factor that on the one hand promotes aging and on the other hinders cell death.

## **6.** **NFIL3 Influences Cellular Survival**

Programmed cell death is utilized throughout development and during homeostatic programs in tissues. For example, 50% of neurons undergo apoptosis during development^67^. NFIL3 has emerged as a survival factor that hinders the induction of apoptosis in numerous settings from B-cells to motor neurons^2,19,68^. Nfil3 was first shown to hinder apoptosis in murine pro-B lymphocytes which normally require the addition of IL3 (Interleukin 3) to the media for cellular survival^19^. IL3 binds to its cognate receptor to activate Ras as well as (B-cell lymphoma-extra large) Bcl-XL thereby mediating survival^18^. The removal of IL3 leads to cell death, which can be rescued by the exogenous expression of anti-apoptotic factors such as Bcl2, Bcl-XL and c-Myc^18^. Nfil3 is thought to have an important role in mediating the IL3 survival signal^19^. *Nfil3* transcription is strongly induced by IL3 in a mechanism that involves the Ras pathway (activates GATA1 which binds to and activates *NFIL3*) and the Phosphatidylinositol 3 kinase (PI3K) pathway in pro-B cells^18,19,69,70^. Conversely, IL3 removal leads to a rapid loss in *Nfil3* expression^19^. Exogenous *Nfil3* expression strongly hinders IL3 deprivation induced cell death in FL5.12 murine pro-B lymphocytes^19^. In BAF-3 pro-B lymphocytes, Nfil3 was shown to rescue viability upon IL3 deprivation^18,19^, but not upon Interleukin 6 (IL6) deprivation^71^. Aside from the interleukin utilized in these rescue experiments (IL3 versus IL6), another key difference was that CD8 was exogenously expressed in the successful rescue experiments, which may have promoted the Nfil3 survival signal^18,19,71^. Loss-of-function experiments also indicate that Nfil3 is a survival factor in pro-B lymphocytes. Specifically, the expression of dominant negative NFIL3 (contains a mutated basic region that fails to bind DNA, but forms heterodimers with wild-type NFIL3) antagonized the ability of IL3 to promote cellular survival^18^. Higher amounts of IL3 were required to maintain Baf-3 pro-B cells in the presence of dominant-negative-NFIL3. Thus, numerous studies indicate that Nfil3 promotes IL3 mediated survival.

In addition to pro-B cells, Nfil3 also impacts the survival of motor neurons. During embryonic development more than half of the motor neurons produced undergo programmed cell death^67^. Nfil3 is highly expressed *in vivo* in embryonic rat and chicken motor neurons that survive developmental pruning^68^. Gain-of-function experiments showed that exogenous *Nfil3* hindered motor neuron cell death upon trophic factor deprivation^68^. In addition to this, Fas ligand (FasL) induced motor neuron death was completely blocked by exogenous *Nfil3*^68^. Gain-of-function experiments were also performed with chicken embryos. Specifically, E2.5 embryos were electroporated with *NFIL3*, which led to the survival of 45% more motor neurons^68^. These results highlight the importance of NFIL3 in neuronal programmed cell death. It will be exciting to determine whether NFIL3 impacts apoptosis during additional developmental processes, and particularly those that involve pruning.

Although many studies have shown an anti-apoptotic affect for Nfil3, this may not be the case for all physiologic settings. In glucocorticoid (GC) signal transduction, Nfil3 may induce cell death^3,72,^. The glucocorticoid receptor (GR) is normally regulated by a negative feedback mechanism upon activation; the GR mRNA and protein are down-regulated after GC addition^3^. However, in certain GC treated murine leukemic T cell lines, GR activation is not attenuated leading to glucocorticoid induced cell death^3,72,73^. The *Nfil3* gene is induced under these conditions and is thought to be part of the mechanism that activates apoptosis. The knockdown of *Nfil3* by RNA interference in CTLL-2 cytotoxic T lymphocyte cells partially rescued GC induced apoptosis^3^. Thus, Nfil3 regulates cell death in a context dependent manner.

## **7.** **NFIL3 in Cancer**

In line with its ability to hinder cell death, NFIL3 has emerged as a novel survival factor in cancer. *NFIL3* was found highly expressed in a number of poor prognosis cancers such as glioblastoma multiforme and basal like breast cancer. *NFIL3* expression in breast cancer was found strikingly associated with poor prognosis by Kaplan Meier survival analysis^2^. Functionally, NFIL3 binds to and represses pro-apoptotic genes such as *TRAIL* to hinder the induction of apoptosis in cancer cells. The diminishment of NFIL3 by RNA interference led to cell death in the BT549, MDA-MB-468 and U87MG cancer cell lines^2^. Conversely, *NFIL3* overexpression hindered H_2_O_2_ induced apoptosis in these same cancer cell lines^2^. On a mechanistic level, NFIL3 acted at least in part to physically block FOXO1 (Forkhead box O1) recruitment to apoptosis inducing genes by binding to promoter elements that have adjacent FOXO and NFIL3 consensus sites^2^. It is important to mention that NFIL3 did not block FOXO1 recruitment to all target genes, but just to a subset of genes involved in tumor suppression^2^. This data suggested that nuclear FOXO1 may still trans-activate non-NFIL3 target genes and that it may even promote cancer in this setting. Therefore, NFIL3 may physically block the ability of FOXO1 to induce cell death genes, so that FOXO1 can direct pro-oncogenic transcriptional programs without killing cells. At least two other recent studies have also found that nuclear FOXO may have pro-oncogenic roles. First, FOXO1 was found to be mutated to a presumably activated form in 8.6% of DLBCL (Diffuse large non-B-cell lymphoma); the FOXO1 mutations in this setting were strikingly associated with poor prognosis^74^. The FOXO1 mutations that were discovered in DLBCL clustered to two regions within the FOXO1 protein. One of the regions alters residues in the DNA binding domain, whereas the other affected region is proximal to Threonine 24 (T24), which is phosphorylated by AKT on the PI3K pathway^74,75^. T24 phosphorylation on FOXO1 promotes its interaction with the scaffolding protein 14-3-3, leading to cytoplasmic localization^75^. Interestingly, the M1L, R19Q, R21C, and T24 mutant forms of FOXO1 (found in DLBCL) failed to be phosphorylated on T24, failed to interact with 14-3-3 and were retained in the nucleus to presumably drive gene expression, suggesting a pro-oncogenic role for nuclear FOXO1 in this setting^74^. FOXO factors were also discovered to promote the development of AML in an MLL-AF9 murine model for leukemia^76^. In this model the loss of all three FOXO factors led to a significantly longer latency for the development of AML with a 16 fold reduction in leukemia initiating cells. It will be critical to determine whether NFIL3 is required in DLBCLs that contain activated FOXO1 and the MLL-AF9 cancers in order to block the activation of cell death by FOXO factors. The full spectrum of NFIL3 regulated transcriptional programs in cancer have yet to be elucidated. Most likely there will only be partial overlap with its output and the FOXO-induced cell death genes.

In addition to its ability to block FOXO recruitment to cell death genes, NFIL3 may also block the recruitment of Proline Acid Rich (PAR) transcription factors to pro-apoptotic genes in colon cancer^77^. NFIL3 was found to repress the pro-apoptotic, BH3-only gene *BGL-GS *(also known as* BCL2-like 14*) in colon cancer cells^77^. The BCL-GS protein binds to Bcl-XL to promote apoptosis^77^. In contrast, the PAR proteins: DBP, TEF (Thyrotroph Embryonic Factor) and HLF (Hepatic Leukemia Factor) can induce a *BCL-GS* promoter-driven reporter gene in cancer cell lines^77^. The *BGL-GS* gene was repressed by NFIL3 and activated by PAR bZIP factors by regulation through the same consensus site. This study also showed that TEF binding to the endogenous *BCL-GS* gene was induced by cisplatin and etoposide treatment^77^. Thus, NFIL3 may block the recruitment of bZIP transcriptional activators to pro-apoptotic genes to potentially hinder chemotherapeutic response. In addition to colon cancer, NFIL3 may have a role in esophageal cancer as irradiation of esophageal cancer cell lines leads to a loss in *NFIL3* expression^78^. Delineating the full breadth of NFIL3 directed transcriptional programs such as those that counter FOXO and PAR transcription factors will be essential for understanding its contribution to cancer.

## **8.** **Nfil3 in Neuronal Regeneration**

Neuronal regeneration in dorsal root ganglion cells is driven by transcriptional programs that include Nfil3-mediated repression of regeneration associated genes^7,13^. Nfil3 is overexpressed in regenerating DRG neurons *in vivo*, but has a negative impact on neurite outgrowth^13^. Knockdown of *Nfil3* by siRNA and expression of dominant negative NFIL3 induced neurite outgrowth in primary adult rat DRG neurons^13^. The ability of NFIL3 to compete for access to consensus promoter sequences is becoming a recurring theme for its action in signaling circuits. In neurons, Nfil3 appears to compete for access to sites shared with cAMP-response element binding protein (CREB) and CCAAT/Enhancer Binding Protein (CEBPb) to regulate transcriptional programs during neuronal regeneration^7,13^. Nfil3 resides within a feedback loop that starts with cAMP-induced phosphorylation of CREB. Next, phosphorylated CREB activates *Nfil3* expression, which represses CREB target genes as well as Cebpb targets by competing with transcriptional activators for access to target genes. At the same time, Nfil3 binds to its own promoter to repress its own expression^7,13^. This creates a highly regulated pulse of cAMP signaling in neurons. Of note, Nfil3 expression was specifically found in neurons that were able to regenerate, suggesting that it had a role in this process^13^. The experimental evidence points to a role for Nfil3 as a component that may promote a greater signaling sensitivity by being an attenuating factor. In sum, CREB activates transcriptional programs including the *Nfil3* repressor which acts to attenuate the initial signal. In this manner, only a pulse of signal occurs instead of a sustained signal. It will be exciting to determine whether this type of feedback circuitry comes into play in additional Nfil3-mediated signaling pathways.

## **9.** **Nfil3 in Osteoblast Signal Transduction****

*Nfil3* was identified as a target of parathyroid hormone (PTH) in murine osteoblasts by representational display analysis^15^. The addition of PTH to primary mouse osteoblasts rapidly induced *Nfil3* transcription in a manner that did not require new protein synthesis^15^. PTH treatment of osteoblasts induced Nfil3 binding to DNA probes that contained Nfil3 binding sites; this binding could be specifically super-shifted by NFIL3 antibody^15^. However, NFIL3 antibody was unable to super-shift the all PTH induced complexes that bound to the Nfil3 consensus site. Querying TRANSFAC (TRANScription FACtor database) revealed that the Nfil3 DNA binding element closely resembled the Cebpb binding site and it was known that PTH also induced Cebpb expression. Further experiments revealed that CEBPb antibody could super-shift the additional PTH-induced complexes^15^. A model was put forth in which Nfil3 and Cebpb compete for binding sites to repress and activate targets respectively in response to PTH. The signaling downstream of PTH includes adenylate cyclase and PKA (Protein kinase A), which were shown to be required for the ability of PTH to activate Nfil3 expression^21^. In addition, another activator of PKA, prostaglandin E_2_ (PGE2) was also able to activate Nfil3 expression. Forskolin, a direct activator of adenylate cyclase could also induce Nfil3 expression. Cyclooxygenase 2 (Cox2) is thought be one of the Nfil3 repressed genes downstream of PTH in mouse osteoblasts^21^. Additional Nfil3 targets in murine osteoblasts include *Runx2, Osterix, and Phex*^21,35,36^. These studies provide important clues about Nfil3 signal transduction in osteoblasts and place its action downstream of PKA.

## **10.** **NFIL3-CEBP** **b** **Antagonism: A Recurring Theme**

The ability of Nfil3 to compete for DNA access with the transcriptional activator Cebpβ is becoming a commonly observed phenomenon. Nfil3 was found to repress Cebpβ target genes such as Ptgs2, Pgr and Areg in rat ovaries. Chromatin immunoprecipitation analysis with CEBPβ and NFIL3 specific antibodies revealed that hCG treatment led to Cebpβ promoter recruitment at 6 hours post-treatment, which was followed by increasing levels of Nfil3 promoter recruitment (between hours 6-12 post hCG treatment) accompanied by transcriptional repression of target genes and diminishing Cebpβ recruitment^8^. The recruitment of these factors to target sequences was further confirmed by EMSA^8^. In addition to a role in ovulation, NFIL3 was also found to hinder CEBPβ recruitment to Human Hepatitis B viral genes^22^. The overexpression of NFIL3 led to a loss in hepatitis B virion production^22^. In sum, the ability of NFIL3 to hinder CEBPβ promoter recruitment may have a tremendous impact on many cellular signaling circuits and introduces a new recurring paradigm for NFIL3 action.

## **11.** **Conclusions and Future Directions**

**Figure 3:**
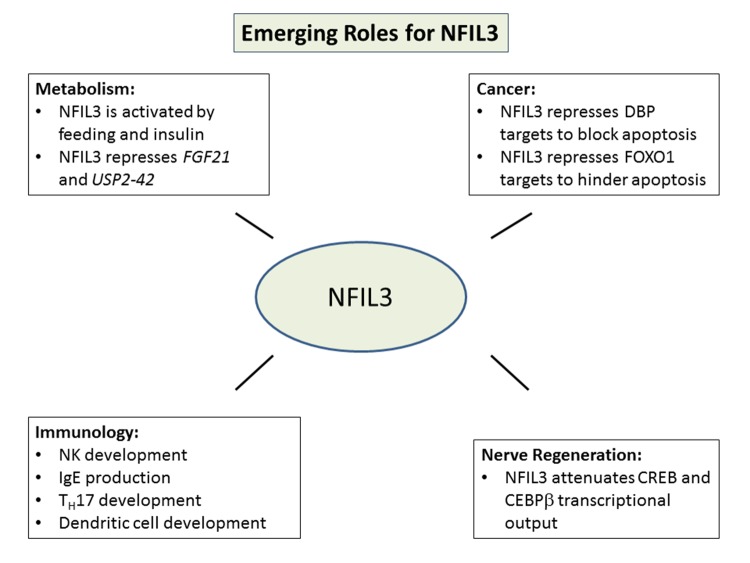
Emerging Cellular Roles for NFIL3 Metabolism, nerve regeneration, cancer development and immunological development have recently been found to be impacted by NFIL3.

NFIL3 is emerging as a key signaling component in a myriad of cellular processes including metabolism, nerve regeneration, immune development and cancer (**Figure 3**). NFIL3 commonly antagonizes the recruitment of transcriptional activators to attenuate a signal. In addition to the well characterized ability of NFIL3 to act in an anti-phase manner to DBP with reference to the circadian rhythm, NFIL3 is recurrently found to act in an antagonistic manner to CEBPb^7,13,15,20^. Nfil3 hinders the recruitment of Cebpb to target genes in motor neurons and osteoblasts. It also appears to hinder CEBPb recruitment during virus production^22^. Very recently, NFIL3 has emerged as a novel factor that might promote human diseases such as diabetes and cancer. Strikingly, the expression of *NFIL3* is significantly associated with poor outcome in cancer^2^. In cancer cells, NFIL3 was found to block FOXO1 access to cell death genes, potentially allowing FOXO1 to drive pro-oncogenic programs, a true paradigm shift for the PI3K pathway^2^. Many of the recent insights into NFIL3 biology have been gleaned from cell-based studies. New frontiers for NFIL3 studies will include more in depth *in vivo* models as well as trying to place NFIL3 into complex signaling cascades. Future research may reveal novel factors for NFIL3 to antagonize as well as mechanisms by which this factor may actually induce transcription as in the case of the *IL3* gene. Finally, the regulation of NFIL3 will be an exciting new frontier for future research and will have broad implications for cellular processes discussed in this review as well as human diseases such as cancer and diabetes.

## KEY POINT

- **NFIL3 transcription factor is emerging as a key signaling component in a myriad of cellular processes, such as: metabolism, nerve regeneration, immune development and cancer.**
